# Bis[4-(dimethyl­amino)­pyridinium] octa­aqua­chloridolanthanum(III) tetra­chloride trihydrate

**DOI:** 10.1107/S1600536812040901

**Published:** 2012-10-03

**Authors:** Meriem Benslimane, Hocine Merazig, Jean-Claude Daran, Ouahida Zeghouan

**Affiliations:** a Unité de Recherche de Chimie de l’Environnement et Moléculaire Structurale, Faculté des Sciences Exactes, Département de Chimie, Université Mentouri de Constantine, 25000 Constantine, Algeria; b Laboratoire de Chimie de Coordination, UPR-CNRS 8241, 205 route de Narbonne, 31077 Toulouse Cedex 4, France

## Abstract

The title organic–inorganic salt, (C_7_H_11_N_2_)_2_[LaCl(H_2_O)_8_]Cl_4_·3H_2_O, consists of two 4-(dimethyl­amino)­pyridinium and one [La(H_2_O)_8_Cl]^2+^ cations, four chloride anions and three solvent water mol­ecules. In the crystal, the various units are connected by N—H⋯Cl, O—H⋯Cl, O—H⋯O and N—H⋯O hydrogen bonds, forming a network of alternating organic and inorganic layers. The 4-(dimethyl­amino)­pyridinium cations stack along the *c* axis, while the inorganic layers lie parallel to the *ac* plane. The chloride anions are located between these entities, forming hydrogen bonds with the NH atom of the pyridinium ions and the water mol­ecules. There are also C—H⋯Cl hydrogen bonds present involving one of the 4-(dimethyl­amino)­pyridinium cations, resulting in the formation of a three-dimensional supra­molecular architecture.

## Related literature

For common applications of organic–inorganic hybrid materials, see: Cui *et al.* (2000 ▶); Lacroix *et al.* (1994 ▶); Chakravarthy & Guloy (1997 ▶). For the crystal structures of compounds involving 4-(dimethyl­amino)­pyridinium, see: Chao *et al.* (1977 ▶); Mayr-Stein & Bolte (2000 ▶); Lo & Ng (2008 ▶, 2009 ▶); Koon *et al.* (2009 ▶). For hydrogen-bond motifs, see: Bernstein *et al.* (1995 ▶).
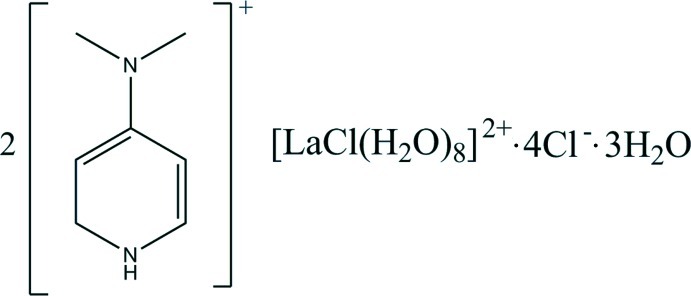


## Experimental

### 

#### Crystal data

(C_7_H_11_N_2_)_2_[LaCl(H_2_O)_8_]Cl_4_·3H_2_O
*M* *_r_* = 760.69
Triclinic, 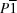

*a* = 9.6741 (4) Å
*b* = 12.6695 (7) Å
*c* = 14.3601 (7) Å
α = 68.354 (5)°
β = 75.273 (4)°
γ = 84.264 (4)°
*V* = 1582.16 (15) Å^3^
*Z* = 2
Mo *K*α radiation
μ = 1.82 mm^−1^
*T* = 180 K
0.43 × 0.28 × 0.08 mm

#### Data collection

Oxford Xcalibur Sapphire1 diffractometer
Absorption correction: multi-scan (*CrysAlis PRO*; Agilent, 2011 ▶) *T*_min_ = 0.548, *T*_max_ = 0.864
33782 measured reflections
7144 independent reflections
6518 reflections with *I* > 2σ(*I*)
*R*_int_ = 0.038

#### Refinement

*R*[*F* ^2^ > 2σ(*F* ^2^)] = 0.023
*wR*(*F* ^2^) = 0.056
*S* = 1.11
7144 reflections
320 parameters
H-atom parameters constrained
Δρ_max_ = 0.76 e Å^−3^
Δρ_min_ = −1.03 e Å^−3^

### 

Data collection: *CrysAlis PRO* (Agilent, 2011 ▶); cell refinement: *CrysAlis PRO*; data reduction: *CrysAlis PRO*; program(s) used to solve structure: *SIR92* (Altomare *et al.*, 1993 ▶); program(s) used to refine structure: *SHELXL97* (Sheldrick, 2008 ▶); molecular graphics: *ORTEPIII* (Burnett & Johnson, 1996 ▶) and *ORTEP-3 for Windows* (Farrugia, 2012 ▶); software used to prepare material for publication: *SHELXL97*.

## Supplementary Material

Crystal structure: contains datablock(s) I, global. DOI: 10.1107/S1600536812040901/su2504sup1.cif

Structure factors: contains datablock(s) I. DOI: 10.1107/S1600536812040901/su2504Isup2.hkl

Additional supplementary materials:  crystallographic information; 3D view; checkCIF report

## Figures and Tables

**Table 1:** Hydrogen-bond geometry (Å, °)

*D*—H⋯*A*	*D*—H	H⋯*A*	*D*⋯*A*	*D*—H⋯*A*
N2—H2*A*⋯Cl5^i^	0.86	2.71	3.314 (3)	129
N2—H2*A*⋯O1*W* ^ii^	0.86	2.24	2.909 (3)	134
N4—H4*A*⋯Cl4^iii^	0.86	2.51	3.213 (2)	140
N4—H4*A*⋯Cl4^iv^	0.86	2.77	3.418 (3)	133
O1—H11⋯Cl2^v^	0.79	2.46	3.2316 (19)	165
O1—H12⋯O1*W* ^vi^	0.78	2.01	2.784 (2)	167
O2*W*—H12*W*⋯Cl3^iii^	0.85	2.38	3.222 (2)	169
O3*W*—H13*W*⋯Cl2^vii^	0.85	2.51	3.293 (2)	153
O2—H21⋯Cl3^viii^	0.85	2.32	3.1537 (17)	166
O1*W*—H21*W*⋯Cl1	0.85	2.31	3.1538 (19)	173
O2—H22⋯Cl4	0.85	2.27	3.1023 (17)	168
O2*W*—H22*W*⋯Cl2	0.85	2.37	3.213 (2)	172
O3*W*—H23*W*⋯Cl2^v^	0.85	2.34	3.193 (2)	176
O3—H31⋯Cl4^viii^	0.84	2.34	3.1471 (17)	160
O3—H32⋯Cl1^vi^	0.84	2.36	3.1413 (16)	157
O4—H41⋯Cl3^viii^	0.84	2.31	3.1459 (17)	173
O4—H42⋯O2*W*	0.85	1.95	2.791 (3)	178
O5—H51⋯Cl1	0.84	2.38	3.1707 (19)	158
O5—H52⋯Cl2	0.85	2.43	3.241 (2)	160
O6—H61⋯Cl4	0.84	2.35	3.1708 (17)	164
O6—H62⋯Cl1	0.85	2.32	3.1250 (16)	158
O7—H71⋯Cl5^v^	0.84	2.46	3.1402 (17)	139
O7—H72⋯Cl1	0.84	2.41	3.2287 (18)	162
O8—H81⋯O3*W*	0.85	1.94	2.786 (3)	172
O8—H82⋯Cl3	0.84	2.48	3.2711 (19)	156
C11—H11*A*⋯Cl3^iii^	0.93	2.80	3.612 (3)	147
C14—H14*B*⋯Cl1	0.96	2.75	3.639 (4)	154

Symmetry codes: (i) 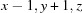; (ii) 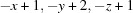; (iii) 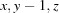; (iv) 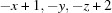; (v) 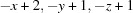; (vi) 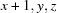; (vii) 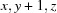; (viii) 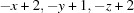.

**Fig. 1.**
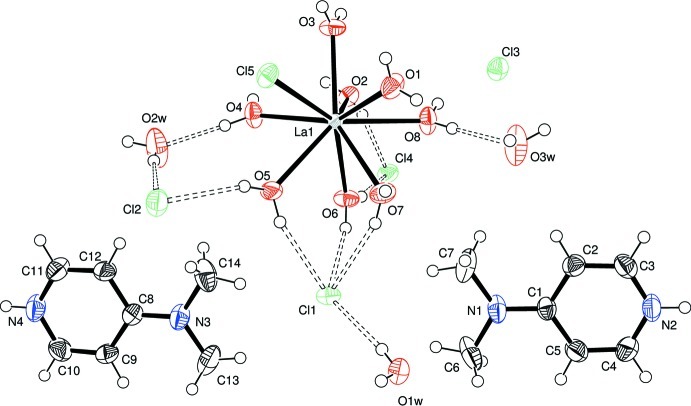
The molecular structure of the title compound, showing the atom-numbering. Displacement ellipsoids are drawn at the 50% probability level. The O—H···Cl hydrogen bonds are shown as double dashed lines.

**Fig. 2.**
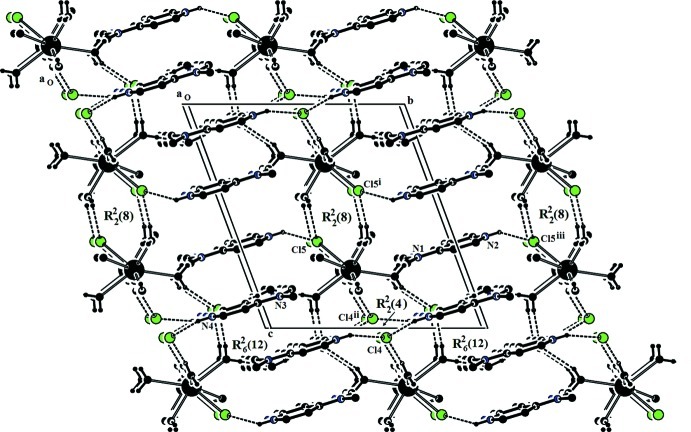
A view along the a axis of the three-dimensional hydrogen-bonded network of the title compound, showing the aggregation of the hydrogen-bonding motifs, R22(4), R22(8) and R62(12). Hydrogen bonds are drawn as dashed lines. [symmetry codes: (i) -x + 2, -y + 1, -z + 1; (ii) -x + 2, -y + 1, -z + 2; (iii) x - 1, y + 1, z].

## supplementary crystallographic information

### Comment

Organic–inorganic hybrid compounds are of great interest because of their special magnetic (Cui *et al.*, 2000), electronic (Lacroix *et al.*, 1994) and optoelectronic properties (Chakravarthy & Guloy, 1997). It is expected that the packing interactions that govern the crystal organization will be influenced by the features of the cations and anions, which in turn will affect specific properties of the solids. The supramolecular networks become especially interesting when the cation and anion can participate in hydrogen-bonding. As part of a study of the effect of cations and anions on the crystal structures of organic–inorganic compounds, we report herein on the crystal structure of the title compound. This type of hybrid material generally exhibits a structure consisting of alternating organic–inorganic layers, characterized by isolated anions as found with other compounds involving 4-(dimethylamino)pyridinium (Chao *et al.*, 1977; Mayr-Stein & Bolte, 2000; Lo and Ng, 2008, 2009; Koon *et al.*, 2009).

The title structure contains three cations, one inorganic [La(H_2_O)_8_Cl]^2+^ cation and two independent monoprotonated 4-(dimethylamino)pyridinium cations, four chloride anions and three water molecules (Fig. 1). Atom La1 is coordinated by eight water molecules with distances ranging from 2.510 (1) to 2.588 (2) Å, and by one chloride ion with La1—Cl5 = 2.8829 (6) Å. The overall structure consists of layers stacked along the c axis. The chloride anions are located between the organic entities forming hydrogen bonds with the NH atoms of the pyridinium ions and the water molecules (Table 1).

Each Cl^-^ anion accepts hydrogen bonds which can be divided into two groups. The first group involves hydrogen bonds linking Cl4^-^ with two organic cations via the pyridinium N4—H4A H atom (Table 1), generating centrosymmetric R_2_^2^(4) motifs (Bernstein *et al.*, 1995) along the c axis at y = 1/2. The second 4-(dimethylamino)pyridinium molecule is linked to one [La(H_2_O)_8_Cl]^2+^ cation through an intermolecular N2—H2A···Cl5^i^ hydrogen bond [symmetry code: (i) *x* - 1, *y* + 1, *z*] which can be described by the graph-set motif D(3). The second type of hydrogen bond, in which the Cl^-^ anion is the acceptor, is a linkage between the water molecules (free and coordinated) and the Cl^-^ anion. The inorganic [La(H_2_O)_8_Cl]^2+^ cations are indirectly linked via Cl^-^ anions through intermolecular O—H···Cl and O—H···O hydrogen bonds generating cycles R_2_^2^(8) and R_6_^2^(12), which connect cationic and anionic entities (Fig. 2 and Table 1).

In the 4-(dimethylamino)pyridinium cations the N—C bond linking the dimethylamino substituent to the pyridinium ring is characteristically short [1.321 (3) and 1.324 (3)Å]. The dimethylamino group lies close to the plane of the pyridinium ring with a dihedral angle, between the pyridinium and the dimethylamine plane (C/N/C atoms), of 3.5 (3) and 2.0 (3)°.

On the structural level, the atomic arrangement of this material consists of a network of alternating organic–inorganic layers. The chloride anions are located between these entities forming hydrogen bonds with the NH atoms of the pyridinium ions and the water molecules. There are also C—H···Cl interactions present (Table 1) involving one of the 4-(dimethylamino)pyridinium cations, which results in the formation of a three-dimensional supramolecular architecture.

### Experimental

4-(Dimethylamino)pyridine (1 mmol, 0.08g) and hydrochloric acid (1M) were added slowly to a solution of LaCl_3_.6H_2_O (1mmol, 0.08g). The mixture was refluxed at 353 K for about 1 h and then cooled to room temperature. Slow evaporation of the solvent at room temperature lead to the formation of colourless plate-like crystals of the title compound.

### Refinement

The H atoms of the coordinated water molecules were located in difference Fourier syntheses and were initially refined using distance restraints: O—H = 0.85 (2) Å, and H···H = 1.40 (2) Å, with *U*_iso_(H) = 1.5*U*_eq_(O). In the final cycles of refinement they were constrained to ride on their parent O atoms. The N-bound H atoms were located in a difference Fourier map but like the C-bound H atoms they were included in calculated positions and treated as riding atoms: N—H = 0.86 Å, C—H = 0.93 and 0.96 Å for CH and CH_3_ H atoms, respectively, with *U*_iso_(H) = 1.5*U*_eq_(C) for the methyl groups and = 1.2*U*_eq_(N,C) for the other H atoms.

### Crystal data

**Table:**

(C_7_H_11_N_2_)_2_[LaCl(H_2_O)_8_]Cl_4_·3H_2_O	*Z* = 2
*M**_r_* = 760.69	*F*(000) = 772
Triclinic, *P*1	*D*_x_ = 1.597 Mg m^−^^3^
Hall symbol: -P 1	Mo *K*α radiation, λ = 0.71073 Å
*a* = 9.6741 (4) Å	Cell parameters from 22004 reflections
*b* = 12.6695 (7) Å	θ = 3.0–28.3°
*c* = 14.3601 (7) Å	µ = 1.82 mm^−^^1^
α = 68.354 (5)°	*T* = 180 K
β = 75.273 (4)°	Plate, colourless
γ = 84.264 (4)°	0.43 × 0.28 × 0.08 mm
*V* = 1582.16 (15) Å^3^	

### Data collection

**Table:**

Oxford Xcalibur Sapphire1 diffractometer	7144 independent reflections
Radiation source: Enhance (Mo) X-ray Source	6518 reflections with *I* > 2σ(*I*)
Graphite monochromator	*R*_int_ = 0.038
Detector resolution: 8.2632 pixels mm^-1^	θ_max_ = 27.5°, θ_min_ = 3.0°
ω scans	*h* = −12→12
Absorption correction: multi-scan (*CrysAlis PRO*; Agilent, 2011)	*k* = −16→16
*T*_min_ = 0.548, *T*_max_ = 0.864	*l* = −18→18
33782 measured reflections	

### Refinement

**Table:**

Refinement on *F*^2^	Primary atom site location: structure-invariant direct methods
Least-squares matrix: full	Secondary atom site location: difference Fourier map
*R*[*F*^2^ > 2σ(*F*^2^)] = 0.023	Hydrogen site location: inferred from neighbouring sites
*wR*(*F*^2^) = 0.056	H-atom parameters constrained
*S* = 1.11	*w* = 1/[σ^2^(*F*_o_^2^) + (0.0257*P*)^2^ + 0.4439*P*] where *P* = (*F*_o_^2^ + 2*F*_c_^2^)/3
7144 reflections	(Δ/σ)_max_ = 0.003
320 parameters	Δρ_max_ = 0.76 e Å^−^^3^
0 restraints	Δρ_min_ = −1.03 e Å^−^^3^

### Special details

**Table:**

Geometry. Bond distances, angles etc. have been calculated using the rounded fractional coordinates. All su's are estimated from the variances of the (full) variance-covariance matrix. The cell esds are taken into account in the estimation of distances, angles and torsion angles
Refinement. Refinement of F^2^ against ALL reflections. The weighted R-factor wR and goodness of fit S are based on F^2^, conventional R-factors R are based on F, with F set to zero for negative F^2^. The threshold expression of F^2^ > 2sigma(F^2^) is used only for calculating R-factors(gt) etc. and is not relevant to the choice of reflections for refinement. R-factors based on F^2^ are statistically about twice as large as those based on F, and R- factors based on ALL data will be even larger.

### Fractional atomic coordinates and isotropic or equivalent isotropic displacement parameters (Å^2^)

**Table:**

	*x*	*y*	*z*	*U*_iso_*/*U*_eq_	
La1	0.98356 (1)	0.46700 (1)	0.74019 (1)	0.0192 (1)	
Cl5	1.19118 (6)	0.36620 (5)	0.61364 (4)	0.0342 (2)	
O1	1.12672 (17)	0.62452 (14)	0.58572 (13)	0.0428 (6)	
O2	0.95865 (16)	0.45114 (13)	0.92689 (11)	0.0297 (5)	
O3	1.21742 (15)	0.45676 (15)	0.78883 (12)	0.0346 (5)	
O4	0.97883 (18)	0.26017 (13)	0.86085 (12)	0.0361 (5)	
O5	0.85068 (17)	0.34195 (15)	0.68566 (14)	0.0436 (6)	
O6	0.71719 (15)	0.46052 (14)	0.83325 (11)	0.0343 (5)	
O7	0.83661 (17)	0.59127 (14)	0.61058 (12)	0.0374 (5)	
O8	0.93133 (19)	0.65745 (14)	0.76941 (12)	0.0383 (6)	
N1	0.4241 (2)	0.81063 (18)	0.68388 (17)	0.0442 (7)	
N2	0.4146 (3)	1.15628 (18)	0.59093 (18)	0.0461 (8)	
C1	0.4218 (2)	0.9227 (2)	0.65305 (18)	0.0322 (7)	
C2	0.5477 (3)	0.9873 (2)	0.62318 (19)	0.0370 (8)	
C3	0.5401 (3)	1.1014 (2)	0.5933 (2)	0.0416 (8)	
C4	0.2929 (3)	1.0995 (2)	0.6179 (2)	0.0479 (9)	
C5	0.2927 (3)	0.9862 (2)	0.6480 (2)	0.0436 (9)	
C6	0.2940 (4)	0.7466 (3)	0.7103 (3)	0.0670 (11)	
C7	0.5568 (4)	0.7456 (2)	0.6892 (3)	0.0618 (11)	
N3	0.4436 (2)	0.07909 (18)	0.85825 (17)	0.0432 (7)	
N4	0.4840 (2)	−0.26628 (19)	0.94889 (18)	0.0473 (8)	
C8	0.4561 (2)	−0.0328 (2)	0.88707 (17)	0.0334 (7)	
C9	0.3378 (2)	−0.1043 (2)	0.9155 (2)	0.0395 (8)	
C10	0.3548 (3)	−0.2174 (2)	0.9445 (2)	0.0465 (9)	
C11	0.5998 (3)	−0.2022 (2)	0.9229 (2)	0.0444 (8)	
C12	0.5908 (2)	−0.0884 (2)	0.89126 (19)	0.0378 (8)	
C13	0.3050 (3)	0.1370 (3)	0.8590 (3)	0.0613 (11)	
C14	0.5691 (4)	0.1508 (3)	0.8259 (3)	0.0609 (11)	
Cl1	0.54269 (5)	0.45268 (5)	0.68027 (4)	0.0344 (2)	
Cl2	0.91549 (7)	0.13843 (5)	0.59452 (5)	0.0433 (2)	
Cl3	0.97602 (7)	0.78867 (5)	0.91759 (4)	0.0388 (2)	
Cl4	0.65967 (6)	0.50633 (5)	1.04257 (4)	0.0340 (2)	
O1W	0.42022 (18)	0.63535 (14)	0.50183 (13)	0.0391 (5)	
O2W	0.9449 (3)	0.06121 (17)	0.82838 (17)	0.0733 (9)	
O3W	0.9016 (3)	0.86973 (17)	0.62175 (16)	0.0676 (8)	
H11	1.09960	0.68050	0.54710	0.0640*	
H12	1.20950	0.61670	0.56780	0.0640*	
H21	0.99110	0.39140	0.96650	0.0450*	
H22	0.88380	0.47160	0.96220	0.0450*	
H31	1.22900	0.46190	0.84310	0.0520*	
H32	1.29400	0.44680	0.74990	0.0520*	
H41	0.99590	0.24250	0.91960	0.0540*	
H42	0.96630	0.19990	0.85180	0.0540*	
H51	0.76890	0.35990	0.67430	0.0650*	
H52	0.88930	0.29500	0.65740	0.0650*	
H61	0.68810	0.48120	0.88450	0.0510*	
H62	0.65100	0.46360	0.80330	0.0510*	
H71	0.87490	0.61120	0.54710	0.0560*	
H72	0.75130	0.57010	0.62470	0.0560*	
H81	0.92450	0.71900	0.72020	0.0570*	
H82	0.95110	0.67060	0.81790	0.0570*	
H2	0.63590	0.95080	0.62430	0.0440*	
H2A	0.41240	1.22910	0.57180	0.0550*	
H3	0.62340	1.14270	0.57390	0.0500*	
H4	0.20700	1.13940	0.61560	0.0570*	
H5	0.20670	0.94860	0.66590	0.0520*	
H6A	0.22750	0.76040	0.76690	0.1010*	
H6B	0.31660	0.66700	0.72960	0.1010*	
H6C	0.25220	0.77020	0.65180	0.1010*	
H7A	0.61200	0.75610	0.62060	0.0920*	
H7B	0.53530	0.66650	0.72600	0.0920*	
H7C	0.61060	0.77130	0.72440	0.0920*	
H4A	0.49240	−0.33900	0.96850	0.0570*	
H9	0.24700	−0.07270	0.91420	0.0470*	
H10	0.27570	−0.26280	0.96190	0.0560*	
H11A	0.68830	−0.23740	0.92680	0.0530*	
H12A	0.67300	−0.04580	0.87200	0.0450*	
H13A	0.25120	0.10850	0.82580	0.0920*	
H13B	0.31940	0.21710	0.82290	0.0920*	
H13C	0.25350	0.12350	0.92890	0.0920*	
H14A	0.61670	0.13040	0.88130	0.0920*	
H14B	0.53970	0.22900	0.80830	0.0920*	
H14C	0.63330	0.14010	0.76690	0.0920*	
H11W	0.43610	0.61070	0.45270	0.0590*	
H21W	0.45310	0.59110	0.55210	0.0590*	
H12W	0.96180	−0.00920	0.85550	0.1100*	
H22W	0.94090	0.07510	0.76670	0.1100*	
H13W	0.92930	0.93010	0.62480	0.1010*	
H23W	0.94670	0.86620	0.56370	0.1010*	

### Atomic displacement parameters (Å^2^)

**Table:**

	*U*^11^	*U*^22^	*U*^33^	*U*^12^	*U*^13^	*U*^23^
La1	0.0199 (1)	0.0190 (1)	0.0202 (1)	0.0010 (1)	−0.0061 (1)	−0.0082 (1)
Cl5	0.0366 (3)	0.0363 (3)	0.0326 (3)	0.0084 (2)	−0.0054 (2)	−0.0195 (3)
O1	0.0333 (9)	0.0315 (10)	0.0442 (10)	0.0023 (7)	0.0006 (7)	0.0014 (8)
O2	0.0320 (8)	0.0342 (9)	0.0248 (7)	0.0070 (7)	−0.0090 (6)	−0.0129 (7)
O3	0.0220 (7)	0.0563 (11)	0.0345 (8)	0.0010 (7)	−0.0080 (6)	−0.0258 (8)
O4	0.0578 (11)	0.0232 (9)	0.0292 (8)	−0.0007 (7)	−0.0161 (7)	−0.0077 (7)
O5	0.0318 (8)	0.0492 (11)	0.0738 (13)	0.0131 (8)	−0.0257 (8)	−0.0438 (10)
O6	0.0251 (7)	0.0533 (11)	0.0307 (8)	0.0001 (7)	−0.0078 (6)	−0.0214 (8)
O7	0.0343 (8)	0.0452 (11)	0.0312 (8)	0.0027 (7)	−0.0141 (7)	−0.0083 (8)
O8	0.0621 (11)	0.0244 (9)	0.0325 (9)	0.0036 (8)	−0.0162 (8)	−0.0124 (7)
N1	0.0510 (13)	0.0267 (12)	0.0516 (13)	−0.0011 (10)	−0.0022 (10)	−0.0167 (10)
N2	0.0626 (15)	0.0242 (11)	0.0532 (14)	0.0006 (10)	−0.0205 (11)	−0.0114 (10)
C1	0.0363 (12)	0.0279 (12)	0.0336 (12)	0.0008 (10)	−0.0043 (9)	−0.0153 (10)
C2	0.0324 (12)	0.0374 (14)	0.0396 (13)	0.0019 (10)	−0.0075 (10)	−0.0130 (11)
C3	0.0449 (14)	0.0366 (15)	0.0413 (14)	−0.0113 (12)	−0.0094 (11)	−0.0093 (12)
C4	0.0444 (15)	0.0405 (16)	0.0643 (18)	0.0127 (12)	−0.0178 (13)	−0.0249 (14)
C5	0.0326 (12)	0.0411 (16)	0.0624 (17)	−0.0019 (11)	−0.0070 (12)	−0.0267 (14)
C6	0.078 (2)	0.0392 (18)	0.082 (2)	−0.0244 (16)	−0.0016 (18)	−0.0250 (17)
C7	0.080 (2)	0.0334 (16)	0.068 (2)	0.0189 (15)	−0.0171 (17)	−0.0187 (15)
N3	0.0473 (12)	0.0294 (12)	0.0466 (12)	0.0040 (10)	−0.0076 (10)	−0.0096 (10)
N4	0.0484 (13)	0.0284 (12)	0.0572 (14)	0.0009 (10)	−0.0025 (11)	−0.0133 (11)
C8	0.0348 (12)	0.0335 (13)	0.0300 (11)	0.0025 (10)	−0.0070 (9)	−0.0102 (10)
C9	0.0262 (11)	0.0425 (15)	0.0491 (15)	0.0028 (10)	−0.0077 (10)	−0.0170 (12)
C10	0.0385 (14)	0.0438 (17)	0.0563 (17)	−0.0104 (12)	−0.0038 (12)	−0.0193 (14)
C11	0.0366 (13)	0.0414 (16)	0.0491 (15)	0.0077 (11)	−0.0074 (11)	−0.0130 (13)
C12	0.0267 (11)	0.0382 (15)	0.0451 (14)	−0.0026 (10)	−0.0058 (10)	−0.0123 (12)
C13	0.072 (2)	0.0458 (18)	0.067 (2)	0.0261 (16)	−0.0248 (17)	−0.0222 (16)
C14	0.076 (2)	0.0347 (17)	0.0626 (19)	−0.0163 (15)	−0.0038 (16)	−0.0109 (15)
Cl1	0.0247 (2)	0.0445 (3)	0.0366 (3)	0.0009 (2)	−0.0099 (2)	−0.0158 (3)
Cl2	0.0529 (4)	0.0348 (3)	0.0459 (3)	0.0063 (3)	−0.0157 (3)	−0.0177 (3)
Cl3	0.0485 (3)	0.0358 (3)	0.0342 (3)	0.0040 (3)	−0.0174 (3)	−0.0110 (3)
Cl4	0.0317 (3)	0.0403 (3)	0.0372 (3)	0.0066 (2)	−0.0128 (2)	−0.0208 (3)
O1W	0.0455 (10)	0.0304 (9)	0.0404 (9)	0.0044 (7)	−0.0106 (8)	−0.0125 (8)
O2W	0.138 (2)	0.0342 (12)	0.0592 (13)	0.0006 (13)	−0.0433 (14)	−0.0167 (10)
O3W	0.1061 (18)	0.0352 (12)	0.0511 (12)	0.0020 (11)	−0.0040 (12)	−0.0139 (10)

### Geometric parameters (Å, º)

**Table:**

La1—Cl5	2.8829 (6)	O1W—H11W	0.8500
La1—O1	2.5585 (17)	O1W—H21W	0.8500
La1—O2	2.5632 (15)	N4—H4A	0.8600
La1—O3	2.5101 (15)	O2W—H22W	0.8500
La1—O4	2.5505 (17)	O2W—H12W	0.8500
La1—O5	2.5710 (19)	O3W—H13W	0.8500
La1—O6	2.5775 (15)	O3W—H23W	0.8500
La1—O7	2.5885 (17)	C1—C2	1.418 (4)
La1—O8	2.5786 (19)	C1—C5	1.419 (4)
O1—H11	0.7900	C2—C3	1.348 (4)
O1—H12	0.7800	C4—C5	1.338 (4)
O2—H21	0.8500	C2—H2	0.9300
O2—H22	0.8500	C3—H3	0.9300
O3—H31	0.8400	C4—H4	0.9300
O3—H32	0.8400	C5—H5	0.9300
O4—H41	0.8400	C6—H6B	0.9600
O4—H42	0.8500	C6—H6C	0.9600
O5—H51	0.8400	C6—H6A	0.9600
O5—H52	0.8500	C7—H7C	0.9600
O6—H61	0.8400	C7—H7A	0.9600
O6—H62	0.8500	C7—H7B	0.9600
O7—H71	0.8400	C8—C9	1.412 (3)
O7—H72	0.8400	C8—C12	1.422 (3)
O8—H81	0.8500	C9—C10	1.342 (4)
O8—H82	0.8400	C11—C12	1.343 (4)
N1—C1	1.322 (4)	C9—H9	0.9300
N1—C6	1.456 (5)	C10—H10	0.9300
N1—C7	1.458 (4)	C11—H11A	0.9300
N2—C3	1.339 (4)	C12—H12A	0.9300
N2—C4	1.336 (4)	C13—H13B	0.9600
N2—H2A	0.8600	C13—H13A	0.9600
N3—C8	1.324 (4)	C13—H13C	0.9600
N3—C14	1.462 (5)	C14—H14C	0.9600
N3—C13	1.462 (4)	C14—H14A	0.9600
N4—C10	1.344 (4)	C14—H14B	0.9600
N4—C11	1.341 (4)		
			
Cl5—La1—O1	70.83 (4)	C4—N2—H2A	120.00
Cl5—La1—O2	130.08 (4)	C8—N3—C14	121.1 (2)
Cl5—La1—O3	72.34 (4)	C13—N3—C14	116.7 (3)
Cl5—La1—O4	78.77 (4)	C8—N3—C13	122.3 (2)
Cl5—La1—O5	71.63 (4)	C10—N4—C11	120.3 (3)
Cl5—La1—O6	142.42 (4)	H11W—O1W—H21W	112.00
Cl5—La1—O7	101.27 (4)	C11—N4—H4A	120.00
Cl5—La1—O8	139.80 (4)	C10—N4—H4A	120.00
O1—La1—O2	122.67 (5)	H12W—O2W—H22W	108.00
O1—La1—O3	77.88 (5)	H13W—O3W—H23W	107.00
O1—La1—O4	146.68 (6)	C2—C1—C5	115.6 (2)
O1—La1—O5	112.45 (6)	N1—C1—C2	122.3 (2)
O1—La1—O6	130.10 (5)	N1—C1—C5	122.0 (2)
O1—La1—O7	65.71 (5)	C1—C2—C3	120.3 (3)
O1—La1—O8	70.66 (6)	N2—C3—C2	121.1 (3)
O2—La1—O3	65.87 (5)	N2—C4—C5	121.1 (3)
O2—La1—O4	68.56 (5)	C1—C5—C4	120.9 (3)
O2—La1—O5	124.62 (6)	C3—C2—H2	120.00
O2—La1—O6	70.04 (5)	C1—C2—H2	120.00
O2—La1—O7	128.53 (5)	C2—C3—H3	119.00
O2—La1—O8	65.90 (5)	N2—C3—H3	119.00
O3—La1—O4	80.24 (6)	N2—C4—H4	119.00
O3—La1—O5	135.98 (6)	C5—C4—H4	119.00
O3—La1—O6	135.89 (5)	C4—C5—H5	119.00
O3—La1—O7	142.78 (6)	C1—C5—H5	120.00
O3—La1—O8	88.74 (6)	H6A—C6—H6C	109.00
O4—La1—O5	68.67 (6)	N1—C6—H6B	109.00
O4—La1—O6	82.76 (6)	N1—C6—H6A	109.00
O4—La1—O7	135.63 (6)	H6A—C6—H6B	109.00
O4—La1—O8	133.68 (5)	N1—C6—H6C	109.00
O5—La1—O6	71.22 (6)	H6B—C6—H6C	109.00
O5—La1—O7	69.44 (6)	H7B—C7—H7C	109.00
O5—La1—O8	135.28 (6)	N1—C7—H7A	109.00
O6—La1—O7	70.55 (5)	N1—C7—H7B	109.00
O6—La1—O8	74.49 (6)	H7A—C7—H7B	109.00
O7—La1—O8	72.67 (6)	H7A—C7—H7C	109.00
La1—O1—H11	130.00	N1—C7—H7C	110.00
La1—O1—H12	119.00	N3—C8—C9	122.6 (2)
H11—O1—H12	111.00	N3—C8—C12	121.6 (2)
La1—O2—H21	117.00	C9—C8—C12	115.8 (2)
La1—O2—H22	123.00	C8—C9—C10	120.8 (2)
H21—O2—H22	108.00	N4—C10—C9	121.3 (3)
La1—O3—H31	126.00	N4—C11—C12	121.5 (3)
La1—O3—H32	121.00	C8—C12—C11	120.3 (2)
H31—O3—H32	113.00	C10—C9—H9	120.00
La1—O4—H41	121.00	C8—C9—H9	120.00
La1—O4—H42	131.00	N4—C10—H10	119.00
H41—O4—H42	109.00	C9—C10—H10	119.00
La1—O5—H51	121.00	N4—C11—H11A	119.00
La1—O5—H52	126.00	C12—C11—H11A	119.00
H51—O5—H52	110.00	C11—C12—H12A	120.00
La1—O6—H61	122.00	C8—C12—H12A	120.00
La1—O6—H62	122.00	N3—C13—H13B	109.00
H61—O6—H62	112.00	H13A—C13—H13C	109.00
La1—O7—H71	119.00	N3—C13—H13C	109.00
La1—O7—H72	114.00	H13A—C13—H13B	110.00
H71—O7—H72	112.00	N3—C13—H13A	110.00
La1—O8—H81	121.00	H13B—C13—H13C	109.00
La1—O8—H82	124.00	N3—C14—H14C	110.00
H81—O8—H82	111.00	H14A—C14—H14C	109.00
C1—N1—C6	121.2 (2)	H14B—C14—H14C	109.00
C1—N1—C7	121.9 (2)	H14A—C14—H14B	109.00
C6—N1—C7	116.8 (3)	N3—C14—H14A	109.00
C3—N2—C4	121.0 (2)	N3—C14—H14B	109.00
C3—N2—H2A	119.00		
			
C6—N1—C1—C2	−177.6 (3)	C5—C1—C2—C3	0.5 (4)
C7—N1—C1—C2	−0.1 (4)	N1—C1—C2—C3	−179.3 (2)
C6—N1—C1—C5	2.6 (4)	C2—C1—C5—C4	−0.8 (4)
C7—N1—C1—C5	−179.9 (3)	N1—C1—C5—C4	179.1 (2)
C3—N2—C4—C5	0.0 (4)	C1—C2—C3—N2	−0.1 (4)
C4—N2—C3—C2	−0.2 (4)	N2—C4—C5—C1	0.5 (4)
C13—N3—C8—C9	3.4 (4)	C12—C8—C9—C10	0.1 (4)
C13—N3—C8—C12	−176.6 (3)	N3—C8—C9—C10	−179.9 (2)
C14—N3—C8—C12	2.2 (4)	N3—C8—C12—C11	178.6 (2)
C14—N3—C8—C9	−177.9 (3)	C9—C8—C12—C11	−1.4 (3)
C11—N4—C10—C9	−0.8 (4)	C8—C9—C10—N4	1.0 (4)
C10—N4—C11—C12	−0.5 (4)	N4—C11—C12—C8	1.6 (4)

### Hydrogen-bond geometry (Å, º)

**Table:**

*D*—H···*A*	*D*—H	H···*A*	*D*···*A*	*D*—H···*A*
N2—H2*A*···Cl5^i^	0.86	2.71	3.314 (3)	129
N2—H2*A*···O1*W*^ii^	0.86	2.24	2.909 (3)	134
N4—H4*A*···Cl4^iii^	0.86	2.51	3.213 (2)	140
N4—H4*A*···Cl4^iv^	0.86	2.77	3.418 (3)	133
O1—H11···Cl2^v^	0.79	2.46	3.2316 (19)	165
O1—H12···O1*W*^vi^	0.78	2.01	2.784 (2)	167
O2*W*—H12*W*···Cl3^iii^	0.85	2.38	3.222 (2)	169
O3*W*—H13*W*···Cl2^vii^	0.85	2.51	3.293 (2)	153
O2—H21···Cl3^viii^	0.85	2.32	3.1537 (17)	166
O1*W*—H21*W*···Cl1	0.85	2.31	3.1538 (19)	173
O2—H22···Cl4	0.85	2.27	3.1023 (17)	168
O2*W*—H22*W*···Cl2	0.85	2.37	3.213 (2)	172
O3*W*—H23*W*···Cl2^v^	0.85	2.34	3.193 (2)	176
O3—H31···Cl4^viii^	0.84	2.34	3.1471 (17)	160
O3—H32···Cl1^vi^	0.84	2.36	3.1413 (16)	157
O4—H41···Cl3^viii^	0.84	2.31	3.1459 (17)	173
O4—H42···O2*W*	0.85	1.95	2.791 (3)	178
O5—H51···Cl1	0.84	2.38	3.1707 (19)	158
O5—H52···Cl2	0.85	2.43	3.241 (2)	160
O6—H61···Cl4	0.84	2.35	3.1708 (17)	164
O6—H62···Cl1	0.85	2.32	3.1250 (16)	158
O7—H71···Cl5^v^	0.84	2.46	3.1402 (17)	139
O7—H72···Cl1	0.84	2.41	3.2287 (18)	162
O8—H81···O3*W*	0.85	1.94	2.786 (3)	172
O8—H82···Cl3	0.84	2.48	3.2711 (19)	156
C11—H11*A*···Cl3^iii^	0.93	2.80	3.612 (3)	147
C14—H14*B*···Cl1	0.96	2.75	3.639 (4)	154

Symmetry codes: (i) *x*−1, *y*+1, *z*; (ii) −*x*+1, −*y*+2, −*z*+1; (iii) *x*, *y*−1, *z*; (iv) −*x*+1, −*y*, −*z*+2; (v) −*x*+2, −*y*+1, −*z*+1; (vi) *x*+1, *y*, *z*; (vii) *x*, *y*+1, *z*; (viii) −*x*+2, −*y*+1, −*z*+2.
